# Mass screening for hepatitis B and C in Southern Upper Egypt

**DOI:** 10.1186/s12889-019-7640-1

**Published:** 2019-10-22

**Authors:** Gamal Soliman, Mahmoud S. Elzalabany, Tarek Hassanein, F. DeWolfe Miller

**Affiliations:** 1 Tropical Medicine, Gastroenterology and Hepatology Department, Faculty of Medicine, Al-Azhar University, Cairo, Egypt; 2 Internal Medicine Department, Ahmed Maher Teaching Hospital, Cairo, Egypt; 3 Southern California Liver Centers, Coronado, CA USA; 4 John A. Burns School of Medicine, University of Hawaii, Honolulu, HI USA

**Keywords:** Hepatitis C virus, Hepatitis B virus, Mass screening, Epidemiology, Egypt

## Abstract

**Background:**

It is well documented that Egypt has the highest prevalence of hepatitis C virus (HCV) infection in the world. The recent development of highly effective direct acting antiviral drugs (DAAs), has opened the possibility of treating and curing HCV infection in the Egyptian population on a large scale.

**Methods:**

A screening demonstration project was implemented in southern Egypt in and around the city of Luxor. Free screening and if indicated, treatment, was offered to those 16 years or older for anti-HCV antibodies (anti-HCV) and hepatitis B surface antigen (HBsAg) using third generation enzyme immunoassays (Enzygnost® Anti-HCV and HbsAg). Statistical methods included estimation of odds ratios (OR) and 95% confidence intervals (95% CI).

**Results:**

There was a large turnout of 67,042 persons who were screened in a 12-month period starting in June 2016. Thirty-one thousand nine hundred sixty-four males (47.7%) and 35,074 females (52.3%) were screened with a mean age of 43.6 ± 14.3 years. Nine thousand seven hundred one patients (14.5%) were positive for anti-HCV and 2950 (4.4%) for HBsAg. Prevalence of anti-HCV was significantly higher in males than females (19.67% vs.9.73% OR = 2.27; CI 2.2 to 2.4; *p* < 0.001) and the same for HBsAg (6.2% vs. 2.8% OR = 2.3; CI 2.2 to 2.5; *p* < 0.001). The prevalence of anti-HCV was significantly associated with age (*p* < 0.001), ranging from between 1 and 4% in individuals below the age of 40 years, then increased steadily to 42% at age 60 followed by a precipitous decline in age specific prevalence.

**Conclusions:**

The results showed unanticipated patterns in the Luxor area of anti-HCV and HBsAg by age and gender in contrast to previous reports on this unique HCV epidemic in Egypt. Moreover, the level and rate of turnout, cost, and other logistical issues, provided essential information for effective planning, design, and evaluation methods for larger national mass screening and treatment programs.

## Background

HCV is a global health problem [1] and Egypt has the highest HCV prevalence in the world, estimated to be 15% in some studies [2, 3]. This Egyptian HCV epidemic has historic origins; many consider the cause to be injection campaigns carried out by the health authorities in the nineteen sixties and seventies. In these campaigns, intravenous injection of tartar emetic was used to treat rural endemic schistosomiasis. Glass syringes and needles were reportedly reused and improperly sterilized resulting in mass transmission of hepatitis C virus [4]. More recent reports have shown that large historic and concurrent iatrogenic exposures [5–7] contribute to ongoing transmission of HCV in Egypt [8].

HCV infection is essentially asymptomatic or presents with nonspecific symptoms and is often missed and frequently does not present as disease for one or more decades [9]. Egypt has, proportionally, the largest HCV RNA positive asymptomatic population in the world [10].

In response to this issue, the Egyptian National Committee for Control of Viral Hepatitis (NCCVH), initiated a national HCV treatment program in 2007. Between 2007 and 2016, 54 treatment centers were established throughout Egypt. From 2008 to 2014, approximately 360,000 patients were treated with pegylated interferon and ribavirin. Sustained viral response (SVR) rates were low, about 54% [11]. Moreover, the course of treatment was long, expensive, with frequent adverse side effects, and a high dropout rate.

A new era in the clinical management of hepatitis C infection emerged with the development of the new direct acting antiviral agents (DAAs) [11]. In 2014, the NCCVH signed an agreement with the manufacturer of sofosbuvir to provide the drug for nearly 1% of its price in the market. Similar contracts were made with the international manufacturers of other DAAs and local production of these products was established resulting in a decrease in price of generic DAAs. The first group of Egyptian patients started receiving treatment in October 2014 [12].

Added to these efforts, the nonprofit Egyptian presidential Tahya Misr fund was established. The fund supports the development of HCV treatment centers in selected underserved areas in cooperation with the Ministry of Health (MOH). These centers are aimed to be a model of care for HCV infected patients, with state-of-the-art facilities, highly qualified staff and no cost screening and treatment for patients. One of the first centers was built in Southern Egypt (Luxor) and was inaugurated in June 3, 2016, called the Luxor HCV Treatment Center (LHCVT Center).

The new LHCVT Center strategy was proactive and predicated on screening for anti-HCV as opposed to the previous national programs which treated only those presenting with symptoms. Accordingly, the goal was changed to a large-scale HCV screening program which is to identify and treat the asymptomatic population. In large part, the feasibility and success of the LHCVT Center is detailed in this report.

The major resource aims of the LHCVT Center were to assess the level of community turnout, the level of personnel needed, laboratory space and equipment needed for HCV antibodies screening and HCV RNA testing of antibodies positive patients, personnel and space needed for clinical assessment and treatment, and the level of data management and managerial resources necessary. This information is required to rationally prioritize national level screening programs.

More specifically, the aim of this investigation was to determine who was most likely to be anti-HCV positive in a screening population and describe the age and sex specific prevalence of anti-HCV and HBsAg in those individuals based on those who participated in the LHCVT Center screening project. As a point of reference, the results were compared to data on the prevalence of anti-HCV and HBsAg from a national representative sample, published in 2015 [13].

This report presents valuable data and results generated by the LHCVT Center that addressed the above aims essential for efficient screening and important new insights into the local patterns of HCV and HBV in Southern Egypt.

## Methods

Screening, defined in the context of this project, was passive meaning that individuals in the community could, of their own volition, present to the LHCVT Center (or nearby outreach locations) and request to be tested for hepatitis B and C. Testing, and if found positive, diagnosis and treatment with the new DAAs was given free of charge. Standard of care for HBsAg positive patients was also provided at no cost.

A blood sample (10 cc), was drawn by venipuncture from those 16 years old or older into an EDTA vacutainer tube and, if outside the LHCVT Center, transported in a cool box to the center laboratory in Luxor city. Sera were tested for anti-HCV antibodies and HBsAg using third generation enzyme immunoassays (Enzygnost® Anti-HCV and HBsAg, Siemens, Germany). The Enzygnost® Anti-HCV assay has a reported > 99.4% specificity [14].

Because HCV RNA results are not included with the first stage screening results, HCV RNA results were estimated. Estimation was based on the ratio of anti-HCV positivity to HCV RNA positivity from an analysis of the large data sets from the DHS 2008 (*n* = 13,616) and EHIS 2015 (*n* = 26,047) national representative samples [2, 13]. These results were 0.677 and 0.667 respectively, the weighted mean of these two values was 0.673, i.e. 67.3% of those who were anti-HCV positive were estimated as also HCV RNA positive. A systematic review on HCV RNA viremic rate found that the pooled mean estimate, 67.3% was very stable and similar regardless of risk, population or subpopulation, country/subregion, background anti-HCV prevalence, age, or sex [15, 16]. For each age group, HCV RNA prevalence was estimated by multiplying anti-HCV prevalence by the weighted mean of HCV RNA: 0.673. By convention, prevalence here is reported as a percentage.

The LHCVT Center designed and pre-tested structured data recording forms and trained local personnel on how to collect and record data for each participant.

Starting from June until October 2016, more than 10,000 persons presented at the LHCVT Center and were voluntarily screened for hepatitis B and C markers. By the end of May 2017, a total of 71,952 persons were screened for which data were available for 67,042 persons at the time of this analysis. This was achieved by adding additional rural and township settings for screening and specimen collection within the Nile River valley and within 20 km in either direction of Luxor (Latitude 25°38′23.98“N and Longitude 32°39’12.15”E).

Data and statistical analysis were done using Epi Info™ 7.2.1.0 developed by the US Centers for Disease Control and Prevention (CDC) [17]. For prevalence estimates, 95% confidence intervals for proportions were calculated. Associations were estimated by odds ratio (OR) with 95% confidence intervals (CI). Results with a statistical *p* < .05 were considered significant. Logistic regression was used to adjust OR estimates using the Mantel Haenazel procedure to test if crude OR (cOR) were different from stratified adjusted OR (aOR): a test for potential confounders.

## Results

**Fig. 1:**
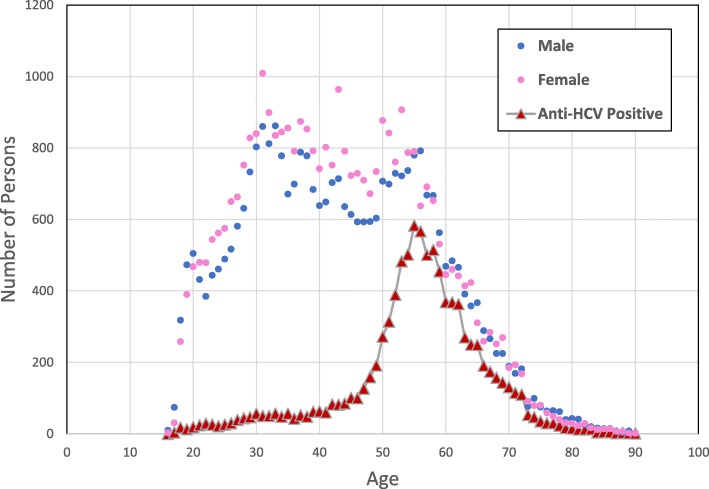
Absolute numbers of persons screened by age and gender and the absolute numbers of persons tested positive for anti-HCV by age. For example, in those 55 years old, 583 persons tested positive, the highest of any age

**Fig. 2:**
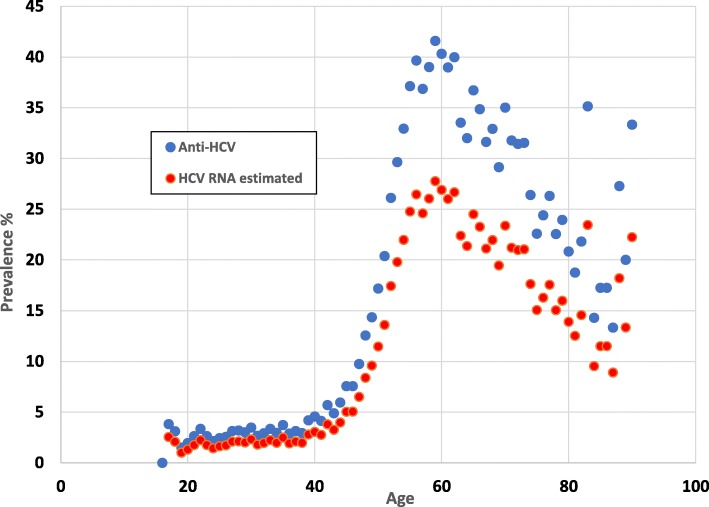
Anti-HCV and estimated HCV RNA prevalence by age (see Methods for estimation method). In those aged 55, the prevalence of anti-HCV was 37.1%. The age specific denominators became increasingly smaller in those over 60, leading to statistical instability in those over 80

Complete screening data for anti-HCV and HBsAg testing of 67,042 persons was available at time of data analysis and included 31,965 males (47.7%) and 35,077 females (52.3%). For age, the mean, median, and interquartile range was 43.6 years, 43 years, and 22 years, respectively. The age structure of the participants, in 5-year age groups, rises sharply from 18 years old to 30 years old and then declines gradually after 55 years old (Fig. 1). The age structure of the absolute number who tested positive for anti-HCV peaks at 55 years old and then sharply declines (Fig. 1). The age specific structure of anti-HCV prevalence and proportionally, HCV RNA prevalence also rises sharply at age 40 and continues to age 60 before sharply declining (Fig. 2).

Overall, there were 9701 persons (14.5%) positive for anti-HCV. Prevalence of anti-HCV was significantly higher in males than females (19.67% vs. 9.73% cOR = 2.27; 95% CI 2.2 to 2.4; *p* < 0.0001). The age adjusted aOR = 2.4 and was not significantly different from the unadjusted estimate (Table 1).

**Table 1:** Prevalence of anti-HCV and HBsAg in the screened population stratified by gender

	Positive	Negative	Total	Prevalence (%)
Anti-HCV Abs				
Female	3413	31,664	35,077	9.73
Male	6288	25,677	31,965	19.67
Total	9701	57,341	67,042	14.47
OR^a^ for Male = 2.27; 95% CI 2.2–2.4				
HBsAg				
Female	965	34,097	35,062	2.75
Male	1982	29,963	31,945	6.20
Total	2947	64,060	67,007	4.40
OR for Male = 2.34; 95% CI 2.2–2.5				
Anti-HCV & HBsAg				
Female	24	35,038	35,062	0.07
Male	83	31,862	31,945	0.26
Total	107	66,900	67,007	0.16
OR for Male = 3.8; 95% CI 2.4–5.99				

^**a**^*OR* Odds ratio and *CI* Confidence Interval

Note that anti-HCV prevalence was much higher in those 60 years old and older, 33.8% (95% CI, 32.9–34.8). This includes 3203 persons who make up 33% of the total anti-HCV positives. The gender specific anti-HCV prevalence was similar to the overall adjusted OR for those less than 60 years old and those 60 years old and older difference and was not significantly different from the crude unadjusted OR (crude OR = 2.27 and adjusted OR = 2.29: 95% CI 2.17 to 2.37 and 2.19 to 2.4 respectively).

There were 2947 persons (4.4%) positive for HBsAg. Similar to anti-HCV, HBsAg prevalence was significantly higher in males versus females (6.2% vs. 2.75% OR = 2.3; 95% CI 2.2 to 2.5; *p* < 0.0001) (Table 1). Age adjusted cOR = 2.4 and was not significantly different from the unadjusted estimate.

**Fig. 3:**
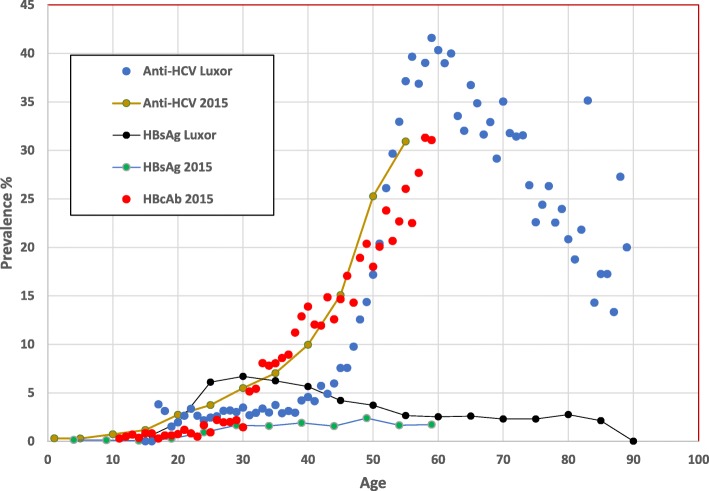
Luxor screening anti-HCV and HBsAg prevalence compared to the 2015 Egyptian Health Issues survey [12] national estimates for anti-HCV, HBcAb, and HBsAg

The age structure of HBsAg prevalence, although much lower than HCV antibody prevalence, has a similar pattern with a steep increase to age 31 (7.7%) followed by a decline to age 60 and then flattens (Fig. 3). A 5-year age group line was generated to show a more distinct pattern over age.

The age specific pattern of HBsAg among those screened in this southern area of Egypt is markedly different and consistently higher than the age specific pattern of HBsAg prevalence estimated by the national EHIS 2015 study (Fig. 3) in which the overall HBsAg prevalence in those 18 years old or older was 1.4%. A complete age specific pattern for the screening results and for the results from the EHIS 2015 study to include anti-HCV, HBcAb, and HBsAg were graphed by prevalence and shown in (Fig. 3). Anti-HCV prevalence in both studies increased rapidly with age. HBcAb prevalence estimated from the EHIS 2015 study closely follows anti-HCV prevalence with similar increases with age.

The prevalence of anti-HCV and HBsAg among those screened, were strongly negatively associated (OR = 0.21, 95% CI = 0.18–0.26) (Table 2). There were 107 persons who were positive for both anti-HCV and HBsAg. Males, relative to females, were associated with being positive for both anti-HCV and HBsAg (OR = 3.8, 95% CI = 2.4–5.99) (Table 1). Moreover, 75 of the 107 were 50 years old or older compared to 32 person less than 50 years old (OR = 4.2, 95% CI = 2.7–6.4).

**Table 2:** Association of Anti-HCV with HBsAg

	HBsAg		
	Positive	Negative	Total
Anti-HCV			
Positive	107	9571	9678
Negative	2840	54,488	57,328
Total	2947	64,059	67,006
OR^a^ for anti-HCV = 0.21; 95% CI = 0.18–0.26			

^**a**^ *OR* Odds ratio and *CI* Confidence Interval

## Discussion

Egypt has established large HCV treatment programs for those with symptomatic disease [18]. We show with data from a unique real-life mass screening program in Southern Egypt that a large number of local individuals clearly desired an opportunity for HCV testing and on being invited to participate, turned out at a high rate (between 5000 to 6000 persons per month). Knowledge of the HCV epidemic in the national and local Egyptian population is well known to be high [2, 3, 12].

That and the opportunity to be treated at no cost by the new highly efficacious DAAs was no doubt a strong motivating factor in the very large turnout from the local community experienced by the project. This well designed and implemented program with strong community support bodes well for a potential goal of treating the entire country in the near future as an achievable reality based on the ultimate objectives of the Tahya Misr project [19].

The screening results showed a high prevalence of anti-HCV (14.5%) in Southern Egypt. This is consistent with the overall prevalence of anti-HCV antibodies in the most recent EHIS 2015 [13] study, which was 9.9%. All screened participants who were positive for anti-HCV were tested for HCV RNA at the Luxor center and if positive received treatment without prioritization [20].

From both national studies, DHS 2008 [2] and EHIS 2015 [13], among those who were positive for anti-HCV, an estimated mean of 67.3% tested positive for HCV RNA regardless of age or gender. There were 9701 persons positive for anti-HCV, which approximates 6529 person positive for HCV RNA (9701 x .673 the estimated proportion with HCV RNA; see Table 1). Persons who screened positive for anti-HCV were subsequently referred for treatment at the Luxor center. In our recent study, we showed that treatment with generic DAAs in asymptomatic patients at the Luxor center had greater than 96% sustained viral response [21].

Anti-HCV prevalence was significantly higher in males and in those older than 40 years which is consistent with results of the previous national surveys. The results strongly suggest that screening focused on those over 40 years old will be a productive strategy for ongoing HCV screening and treatment campaigns in Egypt, or at least in Southern Egypt. This is an important finding. Data from the EHIS 2015 study would have suggested screening starting from a younger age group (Fig. 3). Our observation is reinforced by the data shown in (Fig. 1)**,** in which the absolute largest number of persons who tested positive for anti-HCV, peaked at age 55. In comparison with the two previous national surveys, the peak prevalence of anti-HCV was 44.3, 95%CI 35.9–52.9, at **56** years old in the DHS 2008 study, 36.4, 95%CI 30.2–43.0, at **59** years old in the EIHS 2015 study, and 41.6, 95%CI 38.7–44.5, at **59** years old in this screening study in Luxor.

Also notable was the high HBsAg prevalence of 4.4% overall and 6.2% in males relative to the overall national 1.4% prevalence reported by the EHIS 2015 [13] study. This suggests that HBsAg screening should be recommended for other large screening projects in Egypt, as HBsAg positive patients can develop complications from HCV treatment with DAAs [11]. This finding also suggests a need to improve HBV immunization coverage in this area.

Although the number screened in this study was very large, it was not a representative sample of the local population in Southern Egypt or the city of Luxor. This must be borne in mind when comparing results with nationally representative samples. There was a clear difference in the age specific patterns of anti-HCV antibodies and HBsAg as seen in (Fig. 3). As a footnote, the national studies may have a bias towards more healthy individuals (ill individuals tend to be unavailable or less likely to participate). Nevertheless, our recommendation to recruit older adults, 40 years of age or older, is based on the results of screening for HCV and will likely be more productive and efficient for designing larger national screening programs.

Table 1 showed significant differences in anti-HCV and HBsAg prevalence between genders. This was not seen in the two national studies [2, 13] where the differences between genders were much smaller. The OR is greater than two for both viruses and greater than three for those positive for both anti-HCV and HBsAg. The latter was remarkably more common in those 50 years old and older. Further inquiry may shed light on the association of HBsAg with males.

There are no Egyptian population-based data for anti-HCV in those 60 years old and older. This is not an insignificant group of the population [22]. The level of HCV infection in this older age group was essentially unknown. The overall anti-HCV prevalence in this older age group was greater than the overall prevalence; 33.8% compared to 14.7% respectively. As seen in (Figs. 2 and 3), there is a dramatic decline in anti-HCV prevalence after the age of 60. This precipitous decline generates questions about the epidemiologic history of HCV in Egypt and the natural history of HCV in general. In Egypt, HCV has been iatrogenically transmitted throughout the health care system for decades [3, 6, 7]. Conjecture suggests that over 60 years ago, the Egyptian health care system was much smaller, and percutaneous exposures were less common. There is evidence that the global introduction of glass syringes and re-usable needles iatrogenically spread bloodborne infections globally and was an impetus for the introduction of use only once needles and syringes seen today [23]. Those over 60 years old may have had a much-reduced percutaneous exposure in the past. This remains to be shown.

Another contribution to the anti-HCV prevalence decline in those 60 years old and older may, in part, be attributable to a selection bias known as survival bias [24] that would be more apparent in older age groups. However, additional investigation is needed to address this issue.

We are in the process of investigating possible explanations for the unusual gender difference of prevalence for both viruses, the unanticipated higher prevalence of HBsAg in the Luxor area relative to national data, the odd gender/age distribution of those positive for both anti-HCV and HBsAg, and why both viruses precipitously decline in the older adult age groups.

As progress in screening, treatment, and cure moves towards ridding HCV from Egypt, all efforts must continue to prevent HCV transmission through strict infection control measures in healthcare settings throughout the country [3].

## Conclusions

The results of this large screening project showed unanticipated patterns of anti-HCV and HBsAg by age and gender in contrast to previous reports on this unique HCV epidemic in Egypt. Efficient cost-effective screening for anti-HCV in Egypt should target older age groups. The dramatic decline of anti-HCV prevalence in those 60 years old and older is a unique observation that potentially will shed light on the origins and course of this epidemic in Egypt. The higher than expected prevalence of HBsAg justifies strengthening HBV immunization in southern Egypt and a cautionary advisory for DAA therapy in those HBsAg positive. Overall, the results provide essential information for effective planning, design, and evaluation for future Egyptian national mass HCV screening and treatment programs.

## Supplementary information

**Additional file 1: Table S1a.** Luxor screening total by age, anti-HCV prevalence, and HBsAg prevalence. **Table S1b.** EHIS 2015 total sample by age and sex, anti-HCV prevalence and HBsAg prevalence.

## Data Availability

Data from this screening program are confidential and belong to the Egyptian national Tahya Misr fund. Requests can be made by direct contact with Dr. Gamal Soliman.

## Abbreviations

Anti-HCV: Hepatitis C Virus antibodies
aOR: Adjusted Odds Ratio
CDC: US Centers for Disease Control and Prevention
CI: Confidence intervals
cOR: Crude Odds Ratio
DAAs: Direct acting antiviral agents
DHS: Egyptian Demographic Health Study 2008
EHIS: Egypt Health Issues Survey 2015
HBcAb: Hepatitis B core antibody
HBsAg: Hepatitis B surface antigen
HCV RNA: Hepatitis C Virus ribonucleic acid
LHCVT Center: Luxor HCV Treatment Center
MOH: Egyptian Ministry of Health
NCCVH: Egyptian National Committee for Control of Viral Hepatitis
OR: Odds Ratio
SVR12: Sustained virologic response at 12 weeks
