# Supplementary material for: Mass screening for hepatitis B and C in Southern Upper Egypt
Source: BMC Public Health. 2019 Oct 22;19:1326. doi: 10.1186/s12889-019-7640-1 (PMC6805514; doi:10.1186/s12889-019-7640-1)
Supplement: Supplementary file 1 — Additional file 1: Table S1a. Luxor screening total by age, anti-HCV prevalence, and HBsAg prevalence. Table S1b. EHIS 2015 total sample by age and sex, anti-HCV prevalence and HBsAg prevalence. [file 12889_2019_7640_MOESM1_ESM.docx]

**Table S1a Luxor screening total by age, anti-HCV prevalence, and HBsAg prevalence**

|  | | | | |  |  |  |  |  |  |
| --- | --- | --- | --- | --- | --- | --- | --- | --- | --- | --- |
|  |  |  |  | **Prevalence** | **95% CI** | **95% CI** |  | **Prevalence** | **95% CI** | **95% CI** |
| **Age Group** | **Female** | **Male** | **Total** | **Anti-HCV** | **Lower** | **Upper** |  | **HBsAg** | **Lower** | **Upper** |
| 16-19 | 689 | 877 | 1566 | 2.2 | 1.6 | 3.1 |  | 0.6 | 0.4 | 1.2 |
| 20-24 | 2533 | 2227 | 4760 | 2.5 | 2.1 | 3.0 |  | 1.9 | 1.6 | 2.3 |
| 25-29 | 3468 | 2951 | 6419 | 2.9 | 2.5 | 3.3 |  | 6.1 | 5.5 | 6.7 |
| 30-34 | 4428 | 4115 | 8543 | 3.1 | 2.7 | 3.5 |  | 6.1 | 6.2 | 7.2 |
| 35-39 | 4166 | 3620 | 7786 | 3.4 | 3.0 | 3.8 |  | 6.2 | 5.7 | 6.8 |
| 40-44 | 4051 | 3341 | 7392 | 5.0 | 4.6 | 5.6 |  | 5.6 | 5.1 | 6.2 |
| 45-49 | 3568 | 2998 | 6566 | 10.3 | 9.6 | 11.1 |  | 4.2 | 3.8 | 4.7 |
| 50-54 | 4174 | 3594 | 7768 | 25.2 | 24.3 | 26.2 |  | 3.7 | 3.3 | 4.2 |
| 55-59 | 3303 | 3470 | 6773 | 38.7 | 37.5 | 39.9 |  | 2.7 | 2.3 | 3.1 |
| 60-64 | 2185 | 2168 | 4353 | 37.2 | 35.8 | 38.7 |  | 2.5 | 2.1 | 3.0 |
| 65-69 | 1375 | 1372 | 2747 | 33.3 | 31.6 | 35.1 |  | 2.6 | 2.1 | 3.3 |
| 70-74 | 716 | 716 | 1432 | 31.8 | 29.5 | 34.3 |  | 2.3 | 1.7 | 3.2 |
| 75-79 | 259 | 306 | 565 | 23.9 | 20.6 | 27.6 |  | 2.3 | 1.4 | 3.9 |
| 80-84 | 110 | 146 | 256 | 21.9 | 17.3 | 27.3 |  | 2.8 | 1.1 | 5.6 |
| 85-89 | 44 | 50 | 94 | 18.1 | 11.6 | 27.1 |  | 2.1 | 0.3 | 7.5 |
| 90-95 | 5 | 13 | 18 | 14.8 | 5.9 | 32.5 |  | 0.0 | 0.0 | 0.0 |
| **Total** | **35074** | **31964** | **67038** | **14.5** | **14.2** | **14.7** |  | **4.4** | **4.3** | **4.6** |

**Table S1b *EHIS 2015* total sample by age and sex, anti-HCV prevalence and HBsAg prevalence**

|  |  |  |  | **Prevalence** | **95% CI** | **95% CI** | **Prevalence** | **95% CI** | **95% CI** |
| --- | --- | --- | --- | --- | --- | --- | --- | --- | --- |
| **Age Group** | **Female** | **Male** | **Total** | **Anti-HCV** | **Lower** | **Upper** | **HBsAg** | **Lower** | **Upper** |
| 1-4 | 1725 | 1929 | 3654 | 0.30 | 0.2 | 0.6 | 0.15 | 0.07 | 0.36 |
| 5-9 | 1889 | 1974 | 3863 | 0.28 | 0.2 | 0.5 | 0.11 | 0.04 | 0.29 |
| 10-14 | 1658 | 1703 | 3361 | 0.73 | 0.5 | 1.1 | 0.06 | 0.02 | 0.23 |
| 15-19 | 1407 | 1252 | 2659 | 1.17 | 0.8 | 1.7 | 0.27 | 0.13 | 0.56 |
| 20-24 | 1176 | 882 | 2058 | 2.73 | 2.1 | 3.6 | 0.91 | 0.58 | 1.44 |
| 25-29 | 1472 | 985 | 2457 | 3.73 | 3.0 | 4.6 | 1.65 | 1.21 | 2.25 |
| 30-34 | 1228 | 938 | 2166 | 5.49 | 4.6 | 6.6 | 1.59 | 1.13 | 2.22 |
| 35-39 | 1052 | 875 | 1927 | 7.02 | 5.9 | 8.3 | 1.89 | 1.36 | 2.62 |
| 40-44 | 802 | 722 | 1524 | 9.95 | 8.5 | 11.6 | 1.57 | 1.05 | 2.34 |
| 45-49 | 766 | 657 | 1423 | 15.07 | 13.3 | 17.1 | 2.39 | 1.71 | 3.34 |
| 50-54 | 723 | 685 | 1408 | 25.26 | 23.0 | 27.7 | 1.65 | 1.09 | 2.48 |
| 55-59 | 583 | 466 | 1049 | 30.91 | 28.1 | 33.9 | 1.72 | 1.07 | 2.73 |
| **Total** | **14481** | **13068** | **27549** | **5.59** | **5.3** | **5.9** | **0.91** | **0.81** | **1.04** |
